# Data of insecticide effects of natural compounds against third instar larvae of *Cochliomyia macellaria*

**DOI:** 10.1016/j.dib.2019.104181

**Published:** 2019-07-03

**Authors:** Amanda Chaaban, Erik Nunes Gomes, Vinicius Sobrinho Richardi, Carlos Eduardo Nogueira Martins, Juliana Sperotto Brum, Mário Antônio Navarro-Silva, Cícero Deschamps, Marcelo Beltrão Molento

**Affiliations:** a Laboratory of Parasitic Diseases, Federal University of Parana, UFPR, Curitiba, PR, Brazil; b Department of Veterinary Medicine, Catarinense Federal Institute, IFC, Araquari, SC, Brazil; c Department of Plant Biology, Rutgers, The State University of New Jersey, New Brunswick, NJ, United States; d Department of Plant Sciences, Federal University of Paraná, Curitiba, PR, Brazil; e Morphology and Physiology the Culicidae e Chironomidae, Federal University of Parana, UFPR Curitiba, PR, Brazil; f Laboratory of Veterinary Pathology, Department of Veterinary Medicine, Federal University of Parana, Curitiba, PR, Brazil; g National Institute of Science and Technology, INCT-Livestock, Belo Horizonte, MG, Brazil

**Keywords:** By-product, Ecofriendly, Blowfly

## Abstract

Morphological biomarkers can be used to establish a diagnosis of fly larvae structural damage and toxicity to target cells by biopesticide candidates. Insecticide activity of natural compounds such as *Curcuma longa* essential oil (CLLEO) extracted from leaves, and its major constituent *α*-phellandrene have proven to be a novel biopesticide candidate against third instar larvae (L3) of *Cochliomyia macellaria*. In this way, groups of 20 L3 were placed on filter paper impregnated with different concentrations of CLLEO, from 0.31 to 2.86 μL/cm^2^ and *α-*phellandrene, from 0.29 to 1.47 μL/cm^2^. The extracts were solubilized in ethanol. Data shown in this article is related to the research article “Can an overlooked by-product from turmeric industry be effective for myiasis control?” Chaaban et al., 2019. Data on L3 toxicity was observed after 6 and 24h of contact with both extracts, as well as a marked reduction of L3 movement, color changes in the cuticle and progressive darkening in their body. Major cuticle damage and L3 mortality were reported.

**Table:** Specifications table

Subject area	*Parasitology*
More specific subject area	*Entomology*
Type of data	*Videos*
How data was acquired	*Microscope stereoscopy*
Data format	*Analyzed*
Experimental factors	*Fresh aerial parts of Curcuma longa leaves, a by-product from turmeric, and its major compound α-phellandrene were assessed for insecticidal activity over Cochliomyia macellaria. The assays were performed as described in the companion paper “Can an overlooked by-product from turmeric industry be effective for myiasis control?”**[1]*
Experimental features	*CLLEO extraction and chemical characterization.* *Establishment of C. macellaria colonies for biological assays on laboratory conditions (27 ± 2 °C and 70% relative humidity).* *Contact tests using filter paper impregnated with CLLEO and its major compound α-phellandrene.* *Major cuticular damage and larvae mortality were reported.*
*Data source location*	*City of Araquari, State of Santa Catarina, Brazil; 26°23′ 33.6691″ S and 48° 44′ 18.3336″ W. Details can be seen in the companion paper “Can an overlooked by-product from turmeric industry be effective for myiasis control?”**[1]*
*Data accessibility*	*Data is displayed with the manuscript.*
Related research article	*Chaaban, A., Gomes, E.N., Richardi, V.S., Martins, C.E.N., Brum, J.S., Navarro-Silva, M.A., Deschamps, C., Molento, M.B. Essential oil from Curcuma longa leaves: Can an overlooked by-product from turmeric industry be effective for myiasis control? Industrial Crops and Products**[1]**.*

**Table:** 

**Value of the data** • Research data highlights the insecticide activity of Curcuma longa (Leaves) essential oil, and its is major compound α-phellandrene against third instar larvae of Cochliomyia macellaria, which is a common myiasis infection in livestock. • The data of the use of the by-product from turmeric, as an ecofriendly bioinsecticide highlights a novel natural alternative, which may be beneficial to the welfare of animals and local economies. • The essential oil extracted from the leaves of Curcuma longa and α-phellandrene demonstrates the possible development of a potent insecticide compound. • The data provide a valuable reference for future data collection using the above biopesticide even against other insects that affect livestock and humans.

## 1 Data

The data of this paper involves the experimental analysis regarding the cuticular damage of the natural compounds *CLLEO*, and its major constituent *α*-phellandrene against L3 of *C. macellaria* [1]. L3 from the control group, showed no change in cuticle morphology after 6 and 24h of contact (Video 1a, 1b; Video 2a, 2b). Data of the insecticide effect of the extracts was observed a few hours after contact with the doses of 1.59 and 1.47 μL/cm^2^ of *CLLEO* and *α*-phellandrene, respectively (Video 1c; Video 2c). Moreover, progressive darkening in L3 body, marked reduction of larval movement, color changes in L3 cuticle and death were observed after treatment (Video 1d; Video 2d).

The following is/are the supplementary data related to this article:

Video 1Third instar larvae (L3) of *Cochliomyia macellaria*. a) Control group (ethanol) 6 h after exposure. b) Control group (ethanol) 24 h after exposure. Observe the normal size and movement and regular cuticle color. c) L3 treated with 1.59 μL/cm^2^ of *Curcuma longa* leaves essential oil (CLLEO) 6 h after exposure. Observe the change in the color of L3 body, decrease motility and some dead larvae. d) L3 treated with 1.59 μL/cm^2^ of CLLEO 24 h after exposure. Note the reduction of movements and progressive larval darkening.1

Video 2Third instar larvae (L3) of *Cochliomyia macellaria*. a) Control group (ethanol) 6 h after exposure. b) Control group (ethanol) 24 h after exposure. Observe the normal size and movement and regular cuticle color. c) L3 treated with 1.47 μL/cm^2^ of *α*-phellandrene 6 h after exposure. Observe the change in the color of L3 body, decrease motility and some dead larvae. d) L3 treated with 1.47 μL/cm^2^ of *α*-phellandrene 24 h after exposure. Note the progressive darkening of the body of L3 and a marked reduction of movements.2

## 2 Experimental design, materials, and methods

### 2.1 Plant material, essential oil extraction and chemical characterization

*Curcuma longa* (leaves) used in this work were cultivated at the Medicinal Plants Unit of the Catarinense Federal Institute (IFC), located at 26° 23′ 33.6691″ S and 48° 44′ 18.3336″ W. The location is at 10.6 m above the sea level in the city of Araquari, Santa Catarina, South of Brazil. The plant cultivation, essential oil extraction and chemical characterization were carried as described in the companion paper [1]. *α*-Phellandrene (CAS: 99-83-2) was acquired commercially (Sigma-Aldrich, São Paulo, Brazil) and certified as having ≥99% purity.

### 2.2 Establishment of Cochliomyia macellaria colonies and larval toxicity

Data of the establishment of stock colonies, insects’ identification, mass reproduction, and the protocol for the biological tests were performed as described in the companion paper [1]*.* The toxicity of *CLLEO* and *α*-phellandrene over L3 of *C. macellaria* was performed using groups of 20 L3, which were placed on filter paper that were impregnated with a range of concentrations of *CLLEO* (0.31–2.86 μL/cm^2^) and *α*-phellandrene (0.29–1.47 μL/cm^2^). L3 were put into glass vials containing filter papers (12.56 cm^2^) impregnated with 0.2 mL of EO solution, that were solubilized in ethanol using the protocol described by Chaaban et al. (2017) [2]. The toxicity was evaluated by observing L3 mortality at 6, 24 and 48 h after contact [2], [3]. Total larval mortality (LM) was calculated [2–4] as follows:

LM = (total dead larvae x 100) / total tested larvae

Damages were measured by macroscopic biomarker changes and microscopic lesions using histological sections and scanning electron microscopy in L3 treated with 1.59 μL/cm^2^ of *CLLEO* and 1.47 μL/cm^2^ of *α*-phellandrene, both solubilized in ethanol. The data on these alterations can be observed in the companion paper [1].
